# Safety After Dark: A Privacy Compliant and Real-Time Edge Computing Intelligent Video Analytics for Safer Public Transportation

**DOI:** 10.3390/s24248102

**Published:** 2024-12-19

**Authors:** Johan Barthelemy, Umair Iqbal, Yan Qian, Mehrdad Amirghasemi, Pascal Perez

**Affiliations:** 1 Faculty of Engineering and Information Sciences, University of Wollongong, Wollongong, NSW 2522, Australia; yqian@uow.edu.au; 2 Centre for Geotechnical Science and Engineering, School of Engineering, University of Newcastle, Newcastle, NSW 2308, Australia; umair.iqbal@newcastle.edu.au; 3 Faculty of Business and Law, University of Wollongong, Wollongong, NSW 2522, Australia; mehrdad@uow.edu.au; 4 Australian Urban Research Infrastructure Network (AURIN), University of Melbourne, Melbourne, VIC 3052, Australia; pascal.perez@unimelb.edu.au

**Keywords:** safety, public transport, artificial intelligence (AI), action recognition, computer vision

## Abstract

Public transportation systems play a vital role in modern cities, but they face growing security challenges, particularly related to incidents of violence. Detecting and responding to violence in real time is crucial for ensuring passenger safety and the smooth operation of these transport networks. To address this issue, we propose an advanced artificial intelligence (AI) solution for identifying unsafe behaviours in public transport. The proposed approach employs deep learning action recognition models and utilises technologies like NVIDIA DeepStream SDK, Amazon Web Services (AWS) DirectConnect, local edge computing server, ONNXRuntime and MQTT to accelerate the end-to-end pipeline. The solution captures video streams from remote train stations closed circuit television (CCTV) networks, processes the data in the cloud, applies the action recognition model, and transmits the results to a live web application. A temporal pyramid network (TPN) action recognition model was trained on a newly curated video dataset mixing open-source resources and live simulated trials to identify the unsafe behaviours. The base model was able to achieve a validation accuracy of 93% when trained using open-source dataset samples and was improved to 97% when live simulated dataset was included during the training. The developed AI system was deployed at Wollongong Train Station (NSW, Australia) and showcased impressive accuracy in detecting violence incidents during an 8-week test period, achieving a reliable false-positive (FP) rate of 23%. While the AI correctly identified 30 true-positive incidents, there were 6 cases of false negatives (FNs) where violence incidents were missed during the rainy weather suggesting more data in the training dataset related to bad weather. The AI model’s continuous retraining capability ensures its adaptability to various real-world scenarios, making it a valuable tool for enhancing safety and the overall passenger experience in public transport settings.

## 1. Introduction

Public transport systems (e.g., buses, trains) are the lifeblood of modern cities, providing a convenient and cost-effective means of transportation for millions of commuters every day [1,2]. However, with the growing population density and increasing complexity of urban environments, public transport systems have also become susceptible to safety challenges [3]. One of the most pressing concerns is the occurrence of violence and abuse within these public spaces, which not only jeopardizes passenger safety but also poses significant operational and reputational risks for transport authorities [4,5].

Acts of violence within public transport settings can range from physical altercations, assaults, and vandalism to more severe incidents like armed attacks and harassment [6]. Such incidents not only traumatize passengers but also disrupt the smooth functioning of public transport services, leading to potential delays, service interruptions [7], and increased public fear [8]. Consequently, addressing and mitigating the risks of violence in public transport has become a critical imperative for ensuring the safety and well-being of passengers and staff.

Conventional methods for monitoring and addressing security issues in public transport often rely on human-based operator monitoring videos from closed circuit television (CCTV) [9], which can be labor-intensive, error-prone, and challenging to manage, especially in large and busy transit systems. Human operators may struggle to identify and respond swiftly to incidents in real time, and their effectiveness may be limited by factors such as fatigue and distraction [10]. It should also be noted that victims do not always report abuse or incidents to the security officers [11].

Herein lies the significance of developing an automated end-to-end artificial intelligence (AI) solution for the detection of violence in public transport. AI-based systems offer a transformative approach to public transport security by harnessing the power of computer vision, deep learning, and real-time data analysis. By automating the process of abnormal behaviour detection, AI can enhance the speed, accuracy, and efficiency of security operations, allowing for rapid responses and proactive interventions. The deployment of an AI-driven violence detection system can lead to numerous benefits. First and foremost, it significantly improves passenger safety, fostering an environment where commuters can travel with confidence and peace of mind. Swift detection and response to violent incidents can prevent escalations and potential harm, reducing the overall impact on victims and bystanders. Furthermore, AI-based surveillance systems offer comprehensive coverage, enabling transport authorities to monitor multiple locations and angles simultaneously, thereby supporting the security officers performing the surveillance. The continuous and automated monitoring capabilities ensure that no incident is overlooked, allowing authorities to address security concerns promptly and better understanding where spatio-tempral distributions of the hotspots. Moreover, the implementation of an AI solution for violence detection can optimize resource allocation and operational efficiency. By automating the detection process, transport authorities can allocate their human resources more strategically, focusing on critical intervention and emergency response tasks. To address this problem from an AI perspective, we explore the research questions (RQs) set out in this manuscript:

RQ1: How can real-world simulations enhance data generation for AI models, addressing ethical concerns and improving performance in data-scarce scenarios?
RQ2: What combination of technologies can be used to develop a real-time AI-driven video analytics pipeline for detecting unsafe behaviours in public transport?

We propose an end-to-end AI solution for detecting inappropriate behaviours in public transportation networks by leveraging an existing CCTV infrastructure and state-of-the-art technologies. The video feed from CCTV cameras is securely streamed to a local edge server using Amazon Web Services (AWS) DirectConnect, ensuring efficient and private data handling. To process the video with privacy compliance in real time, we deploy an action recognition model based on the temporal pyramid network (TPN) [12] using a local edge computing server (i.e., a graphical processing unit (GPU)-powered edge server at the SMART Infrastructure Facility to perform on-board computing), which excels at capturing dynamic temporal patterns in video streams without any personal identifiers. ONNXRuntime serves as the inference engine, enabling fast and cross-platform model deployment, while NVIDIA GPUs provide the computational power necessary for handling high-resolution video streams with low latency. The real-time video analytics pipeline is developed using DeepStream SDK [13], optimized for efficient video processing and deep learning integration. For communication between the edge server and the web dashboard, we use MQTT, a lightweight messaging protocol that ensures reliable and timely transmission of inference results. As part of the operational pipeline, the CCTV network captures video, which is securely streamed via AWS DirectConnect to a local edge computing server. There, the trained TPN model is executed for inference using ONNXRuntime, and the resulting outputs are sent to a web dashboard through the MQTT protocol.

The contributions of the research presented in this work are as follows:

1. Development of a new video dataset from open-source resources and live simulated trials to facilitate the training of TPN action recognition model for unsafe behaviour detection at train stations.
2. Development, deployment and validation of a privacy-compliant and end-to-end pilot AI solution for the detection of specific behaviours in real time from CCTV cameras.

## 2. Related Work

There are very few papers published in regard to violence detection in the transport utility, highlighting the nascent nature of this field. This section reviews the existing solutions where computer vision techniques have been applied to detect violent events in transportation contexts, such as buses and trains. This section aims to provide an overview of the progress made thus far and identify the gaps that remain to be addressed.

In 2022, Ciampi et al. [14] introduced the “Bus Violence” dataset, a new benchmark for violence detection in public transport, featuring video clips with simulated violent and non-violent actions under varying conditions. They evaluated several state-of-the-art models pre-trained on general violence datasets and observed that models struggle to generalize to this specific scenario, revealing the domain shift problem. Authors suggested that domain adaptation techniques and unsupervised learning could enhance performance. In 2023, Marteau et al. [15] used deep learning architectures for violence detection in railway environments. Authors performed a domain adaptation on a state-of-the-art architecture trained on a railway dataset and enhanced it by incorporating a recurrent mechanism to process long-term sequences. The results showed promising performance on the selected datasets (i.e., Hockey Fight, Surveillance Camera Fight), and an evaluation on Suburban Train Fight demonstrated that combining a three-dimensional (3D)-CNN and a graph recurrent unit (GRU) achieved a detection accuracy of 72%.

In 2023, Kulkarni and Chakraborty [16] introduced a novel approach to violence detection through a multi-model training framework, leveraging data categorization to address challenges in generalization and model performance. By categorizing violent actions into subsets and training models separately for each, the system used ResNet 3D as the base model to consolidate pre-trained models into a unified network. The proposed method achieved a 2–3% accuracy improvement over state-of-the-art approaches. The findings highlighted the potential of data categorization to simplify model development and enhance learning capabilities, with applications beyond violence detection. Most recently, in 2024, Tsiktsiris et al. [17] proposed a multi-modal abnormal event detection system to enhance passenger safety on public transportation, specifically inside autonomous vehicles. The system integrated RGB, depth, and audio data using a deep learning architecture to detect events like aggression, theft, and vandalism. Experiments, conducted on a custom dataset, showed promising results with an accuracy of 85.1%, outperforming existing methods. The system’s real-time capability was validated in autonomous minibuses, demonstrating its practical applicability. However, challenges like computational efficiency and distinguishing similar events remain.

The reviewed literature highlights the use of deep learning models like 3D-CNN, GRU, ResNet3D and Multimodal approaches for violence detection in public transport systems. While novel datasets such as the Bus Violence dataset and domain adaptation techniques show promise in improving model generalization, the studies reviewed indicate a common limitation: the struggle to apply pre-trained models, often trained on general datasets, to domain-specific scenarios such as public transport. The domain shift problem, evident in the failure of models to generalize across varying conditions, underscores the need for more targeted data collection and domain-adaptation techniques. Moreover, despite the integration of advanced architectures, like 3D-CNN and GRU, issues like occlusions and distinguishing between similar violent actions remain unresolved. While multi-modal systems that combine video, depth, and audio data have shown potential, challenges around real-time performance, computational efficiency, and the accurate differentiation of events persist. Notably, none of the proposed solutions have been fully validated or deployed in real-world, real-time public transport settings.

## 3. The Proposed AI Solution for Unsafe Behaviour Detection

**Figure 1:**
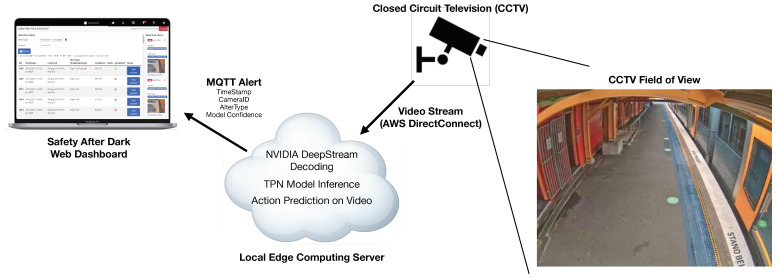
The block diagram representation of the proposed AIoT solution for unsafe behaviour detection.

This section presents the details of the proposed AI solution for unsafe behaviour detection, leveraging the power of deep learning action recognition model. The solution aims to continuously monitor the train station platform using CCTV and securely stream the video to a local edge server using AWS DirectConnect, where the video is processed through the deep learning action recognition model (i.e., TPN) using ONNXRuntime inference engine. The model inference results are then transmitted to a web application using MQTT. Figure 1 shows the block diagram representation of the proposed solution.

**Figure 2:**
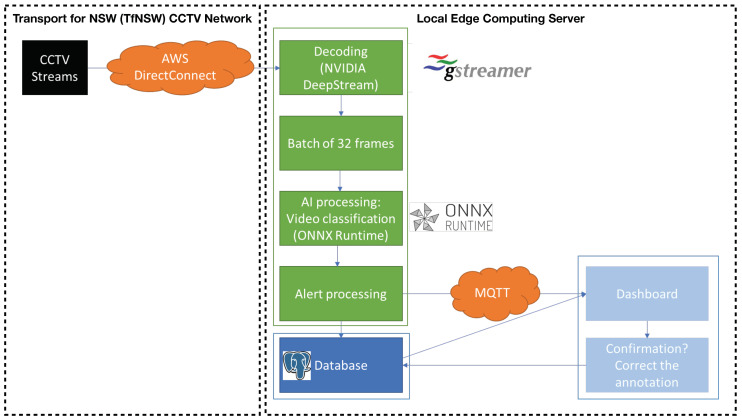
The architecture of the proposed AIoT solution for the unsafe behaviour detection.

The high-level architecture of the solution is designed to enable real-time action recognition from CCTV streams, effectively enhancing the security and surveillance capabilities of Sydney Trains. Illustrated in Figure 2, the system seamlessly integrates various technological components including AWS DirectConnect, local edge server, ONNXRuntime, DeepStream SDK and MQTT, ensuring efficient data flow, processing, and secure communication. To begin, the CCTV streams from Sydney Trains, encoded in a H.264/H.265 format, are securely transmitted to the local edge server through AWS DirectConnect. The ingestion and decoding of these video streams are expertly handled using GStreamer [18], guaranteeing compatibility and optimal video processing. The heart of the system lies in the dedicated local edge server with an AI inference engine, capable of processing each CCTV stream independently. The ONNXRuntime inference engine, powered by the powerful NVIDIA DeepStream 6.0 platform, utilizes NVIDIA GPUs for accelerated AI computations. As the video streams are received, the local edge server performs frame decoding, batching sequences of 32 frames, and initiates AI computations using the efficient ONNXRuntime. When an incident is detected, the system transmits immediate alerts via the secure MQTT protocol to the web application. The technological choices in this solution were carefully selected to ensure efficient, scalable, and secure processing of CCTV streams in real time. AWS DirectConnect was chosen to securely stream the video feeds to the local edge server as it provides a reliable, high-throughput connection with low-latency data transfer, which is crucial for real-time processing. The local edge server was integrated to minimize dependency on cloud-based computing, ensuring faster inference times and reducing bandwidth requirements, as only relevant data (such as alerts) are transmitted. ONNXRuntime was selected as the inference engine due to its cross-platform support and optimization for performance across hardware, enabling the efficient execution of the trained TPN model on the NVIDIA GPUs. Leveraging DeepStream SDK enhances the system’s ability to handle high-throughput video streams, offering optimized tools for video decoding, frame batching, and deep learning model integration. The MQTT protocol was employed for its lightweight, reliable, and low-latency communication, ensuring that alerts are sent in real time to the web application. This study assumed uninterrupted video streaming to maintain focus on developing an effective end-to-end AIoT pipeline for real-time unsafe behaviour detection. The system was designed and tested in an ideal operational environment where video feeds are consistently available. This assumption allowed this study to emphasize the accuracy and performance of action recognition and alerting mechanisms under normal conditions.

**Figure 3:**
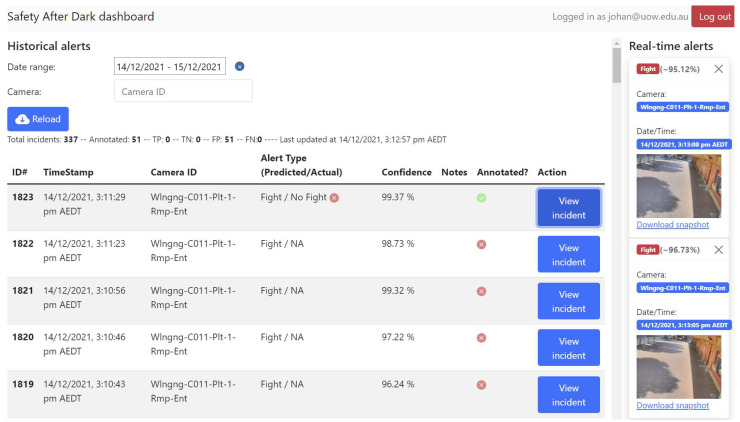
The Safety After Dark web-based front-end.

The web application consisted of two core elements: a front-end implemented using the React framework, shown in Figure 3, and a Node.js back-end hosted on a dedicated virtual machine. The aim was to facilitate real-time monitoring and exploration of detected incidents, as well as to listen to alerts transmitted through MQTT. The user-friendly web-based interface presents actionable information to security personnel, enabling swift responses to potential threats. Additionally, the web application allows for querying a PostgreSQL database deployed in a container, granting access to historical incident data. This empowers users to conduct in-depth analyses, identify patterns, and draw insights for enhanced situational awareness.

To address privacy concerns, the system offers an optional module for automatic face blurring, ensuring individual anonymity while preserving the integrity of the surveillance process. This feature enhances the system’s ethical compliance and fosters public trust. The solution’s modular architecture provides a remarkable advantage, allowing individual components to be easily replaced or relocated without compromising the overall system’s performance and robustness. This flexibility eases maintenance efforts and ensures the system’s adaptability to evolving requirements. In addition, this study employs the TPN model, which operates solely on a global/image scale without extracting any personal identifiers. The TPN model focuses on the movement patterns of pixels across consecutive video frames to recognize actions, ensuring that the analysis remains at an abstract level, solely interpreting motion data. This design inherently respects individual privacy, as no facial or personally identifiable information is derived or processed during action recognition.

For secure communication, alerts transmitted from the AI engine to the web application are protected using TLS 1.3, guaranteeing data privacy and integrity during transmission. As the action recognition model is based on the NVIDIA ecosystem, NVIDIA GPUs are instrumental in enabling efficient and accelerated AI computations, both during the model training and inference stages.

## 4. Temporal Pyramidal Network (TPN)

**Figure 4:**
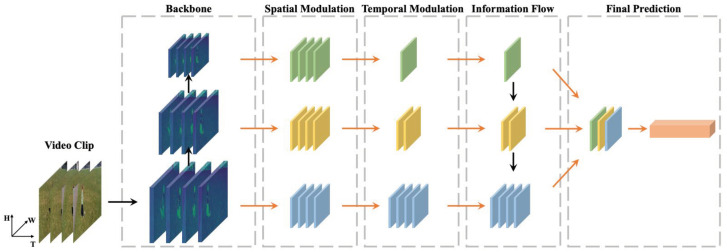
The architecture of the temporal pyramid network (TPN) model for action recognition (image taken from [12]).

TPN is a framework designed for video action recognition, aiming to capture the dynamics and temporal scale of actions, which are crucial for accurate recognition. TPN operates at the feature-level and can be easily integrated into existing two-dimensional (2D) or 3D backbone networks in a plug-and-play manner. The TPN model consists of two essential components: the feature source and the feature aggregation. The feature source involves collecting hierarchical features from the backbone network, where these features have increasing temporal receptive fields from bottom to top. There are two ways to collect these features: single-depth pyramid and multi-depth pyramid [12]. Figure 4 shows the stature of the TPN network.

Let us consider a backbone network that produces hierarchical features $\left\{F_{1},F_{2},\ldots ,F_{M}\right\}$ at different depths, where $F_{i}$ represents the feature at level *i*. These features have increasing temporal receptive fields from bottom to top.

- Single-depth pyramid: Choose a base feature $F_{base}$ at a certain depth and sample along the temporal dimension with different rates $\left\{r_{1},r_{2},\ldots ,r_{M};r_{1}<r_{2}<\ldots <r_{M}\right\}$. The TPN consists of {F(1),F(2),…,F(M)} of sizes {C×T×W×H,…,C×T×W×H}, where *C* is the number of channels, *T* is the temporal dimension, and *W* and *H* are spatial dimensions.
- Multi-depth pyramid: Collect a set of *M* features with increasing depths, resulting in a TPN made of $\left\{F_{1},F_{2},\ldots ,F_{M}\right\}$ of sizes $\left\{C_{1}\times T_{1}\times W_{1}\times H_{1},\ldots ,CM\times TM\times WM\times HM\right\}$, where the dimensions satisfy $\left\{C_{i_{1}}\geq C_{i_{2}},W_{i_{1}}\geq W_{i_{2}},H_{i_{1}}\geq H_{i_{2}};i_{1}<i_{2}\right\}$. This approach captures richer spatial semantics compared to the single-depth pyramid but requires careful feature fusion to ensure correct information flow.

The TPN captures temporal features at different scales (see Figure 4), which is crucial for handling a wide range of action dynamics, from fine-grained details to global patterns. At the lower levels of the pyramid, the TPN focuses on short-term, high-frequency features that correspond to rapid movements, such as hand gestures or quick body posture changes, ensuring that subtle actions are effectively recognized. At higher levels, it captures long-term features that span larger time windows, enabling the model to understand the global context of an action, such as the full sequence of movements in running or dancing. Additionally, the TPN captures intermediate features that represent transitions between different action phases, like moving from walking to running or standing to sitting, helping recognize actions with smooth and gradual changes. The integration of short-term, intermediate, and long-term features across different temporal scales allows the TPN to build a richer understanding of actions, improving its ability to recognize both rapid movements and slower, continuous motions, making it effective for a wide range of action recognition tasks in real-world environments.

To align spatial semantics, a spatial semantic modulation is employed. For each but the top-level feature, a stack of convolutions with level-specific stride is applied, matching its spatial shape and receptive field with the top-level feature. Additionally, an auxiliary classification head is appended to it to receive stronger supervision, leading to enhanced semantics. Traditional action recognition methods generally operate with a fixed temporal receptive field, meaning that they only focus on either short-term or long-term features, but not both. This can lead to suboptimal performance when actions contain both rapid, detailed movements and slower, broader patterns. The TPN, in contrast, captures temporal features at multiple scales, allowing it to efficiently process both fast and slow motions. This is accomplished by aggregating features from different temporal scales in a hierarchical manner, ensuring that the model can adapt to the varying duration of actions in real-world environments.

The overall objective for a backbone network with a TPN becomes

(1) $$L_{total}=L_{CE,o}+\sum \lambda_{i}\times L_{CE,i}$$

$L_{CE,o}$ is the original cross-entropy loss, $L_{CE,i}$ is the loss for the *i*-th auxiliary head and $\lambda_{i}$ represents balancing coefficients.

To control the relative differences of features in terms of temporal scales, a set of hyper-parameters $\left\{\alpha_{1},\alpha_{2},\ldots ,\alpha_{M}\right\}$ is introduced to the TPN for temporal rate modulation. Specifically, $\alpha_{i}$ denotes that after spatial semantic modulation, the updated feature at the *i*-th level will be temporally downsampled by a factor of $\alpha_{i}$ using a parametric sub-net.

For feature aggregation, there are different options (as listed below). The aggregated features from all levels are then rescaled and concatenated for final predictions.

- Top-down flow: Aggregating features from higher-level to lower-level in the pyramid.
- Bottom-up flow: Aggregating features from lower-level to higher-level in the pyramid.
- Lateral flow: Aggregating features at the same level in the pyramid.
- Cascade Flow: Combining top-down and bottom-up flows.
- Parallel Flow: Simultaneously combining top-down and bottom-up flows.

## 5. Experimental Results and Discussions

### 5.1. The Safety After Dark (SAD) Dataset

This section presents the meticulous development of a customized video dataset specifically tailored to facilitate the training of a robust deep learning action recognition model. The dataset comprises a diverse collection of video sources, including publicly available footage and real-world recordings graciously provided by Sydney Trains, ensuring a comprehensive representation of various scenarios encountered in surveillance and video analytics. The dataset was meticulously divided into two distinct categories, each catering to the specific needs of the action recognition model.

**Figure 5:**
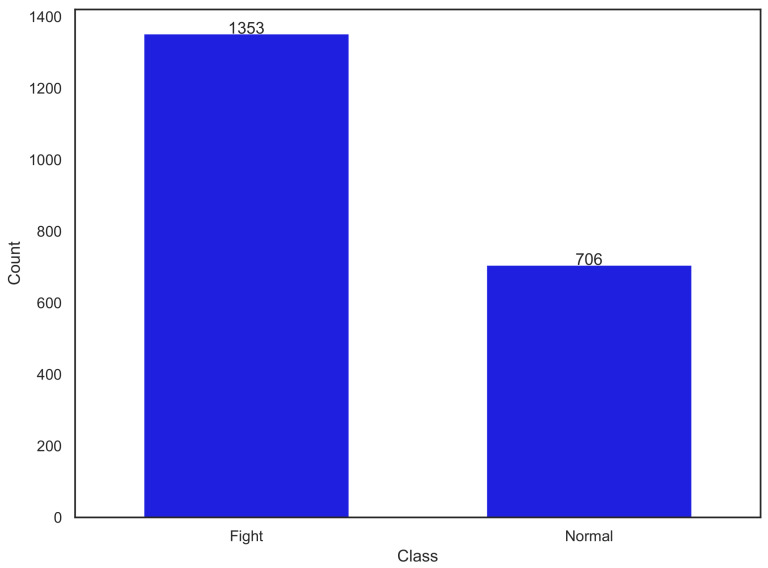
Class-wise distribution of Safety After Dark (SAD) dataset.

**Figure 6:**
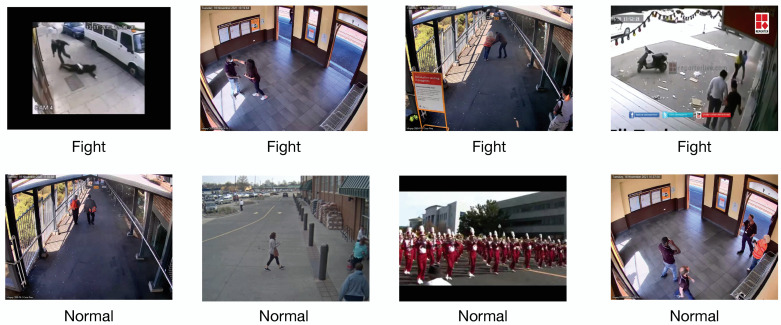
Random dataset snapshots from fighting and normal classes. Top row represent samples from “Fight” class. Bottom row represent samples from “Normal” class with no fighting.

In the first category, we assembled video clips depicting instances of people engaging in physical altercations, sourced from well-established datasets like UCF-Crime [19] and NTU CCTV-Fights Dataset [20]. These video clips were further augmented with real-world footage obtained directly from Sydney Trains, capturing authentic scenes of conflicts in different settings. The inclusion of 677 annotated clips in this category enriches the dataset with challenging and critical action classes, reflecting scenarios of vital interest in safety and security applications. The second category of the dataset was thoughtfully curated to encompass routine activities commonly captured by security cameras. Drawing upon the UCF101—Action Recognition Dataset [21]—we collected video clips portraying individuals performing everyday actions such as walking, sitting, shaking hands, eating, and children playing. To enhance the diversity of non-violent activities, we further integrated clips from the VIRAT Video Dataset [22] and NTU RGB+D Dataset [23,24]. This meticulous curation resulted in a compilation of 1206 video clips, ensuring a comprehensive coverage of routine actions typically encountered in surveillance settings. In total, in the final version of the SAD dataset, there were 2059 clips, 1353 from “Fighting” class while 706 from the “Normal” class. Figure 5 shows the class-wise distribution of the dataset. The class imbalance is evident from the plot with “Fighting” class dominating which may introduce a bias. Figure 6 shows the random snapshots from the dataset belonging to both of the action classes.

To facilitate accurate model training and performance evaluation, each video clip in the dataset was annotated to represent an action class. A standardized format was employed to label each video with its corresponding action class, effectively distinguishing between the “fighting” and “routine activities” categories. This consistent annotation schema ensures a clear understanding of ground truth labels and enables seamless integration into the deep learning action recognition model. Before initiating the model training process, we subjected the video clips to careful preprocessing steps. These steps included resolution normalization and frame rate adjustment to ensure a uniform representation of video data, eliminating potential biases due to variations in video quality. Additionally, the entire dataset was converted to the widely supported MP4 format, enabling easy compatibility with various deep learning frameworks and simplifying the integration process. By providing a meticulously curated and standardized dataset, we contribute significantly to the advancement of action recognition research, fostering the development of more sophisticated video analysis applications across a multitude of domains.

### 5.2. Base Model Training

The base TPN model for fight detection was trained using a patched version of the GluonCV toolkit7 [25]. The training process involved two phases: initial training and retraining. The model’s inference engine was exported and optimized for the ONNXRuntime with the TensorRT backend, utilizing half-precision (FP16) for faster inference. To process the video streams efficiently, the AI inference engine was deployed in a virtual machine equipped with four NVIDIA T4 GPUs, capable of running the model at a constant 20 frames per second (FPS). This hardware setup ensured optimized performance and utilization of the GPUs.

**Figure 7:**
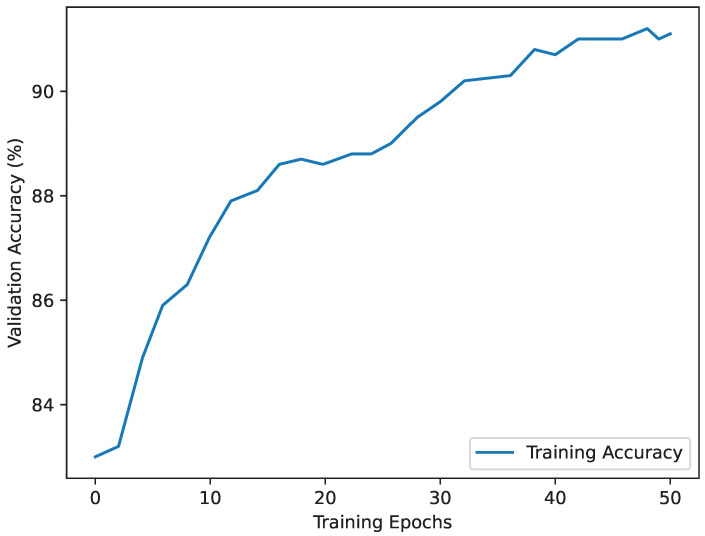
Base TPN model training accuracy curve.

During the base model training, the model was trained for 50 epochs with a batch size of 10 and a learning rate of 0.001. No warm-up training steps were used. To assess its generalization ability, a validation dataset was created, containing images not seen during training, split using a 75/25 ratio from the complete dataset. Regular evaluation of the model’s accuracy was performed on this validation dataset during the training phase. Figure 7 depicts the evolution of the validation accuracy over the 50 epochs. At the 38th epoch, the base AI for fight detection achieved an impressive 93% validation accuracy. Throughout training, regular validation on unseen data was carried out to monitor the model’s ability to generalize and prevent overfitting.

### 5.3. Live Trial and Model Retraining

The live trial of the AI model was a crucial phase in evaluating its performance in a real-world setting. Sydney Trains provided access to the live video feeds from five CCTVs strategically positioned at the Wollongong Train Station. This trial spanned a period of eight weeks in November/December 2021, during which the AI’s ability to detect potential fight incidents was put to the test. Whenever the AI identified a potential fight, the inference engine recorded a five-second video clip of the incident and promptly transmitted an alert. To facilitate the assessment and validation of the alerts generated by the AI, a user-friendly web application was developed. This web-based application provided a comprehensive interface for users to interact with the AI system. Key functionalities of the web application included receiving real-time notifications of incidents, allowing users to confirm or adjust the AI’s predictions based on their assessment, tracking the true-positive (TP) and true-negative (TN) rates for performance evaluation, and enabling users to annotate the incidents based on their feedback.

As actual fight incidents at the Wollongong Train Station were relatively infrequent, the project team planned and conducted two simulation exercises. These simulations took place on the 16th of November and the 6th of December. During these exercises, several University of Wollongong (UOW) staff members played the roles of different actors to enact various fight scenarios in front of the CCTV cameras. The simulation scenarios were designed to encompass different types of fight incidents commonly observed in such public transportation settings. The scenarios included

- One-on-one fights: A person walks, then a second person walks towards the first one and attack the first one. The two persons start to fight.
- One-against-multiple attacks: A person walks, two persons are standing on the way, and then when the first person comes close, the two persons assault the first one.
- Group assaults: A group of persons is chasing and assaulting one individual.
- Clashes between groups: Two groups of persons are colliding.

Each scenario was repeated multiple times with different actors to create a diverse dataset that could represent a wide range of possible real-world scenarios. The first simulation exercise yielded 203 videos, while the second one contributed a larger dataset of 487 videos. This additional data were invaluable for training the AI to recognize and differentiate various fight scenarios effectively.

**Figure 8:**
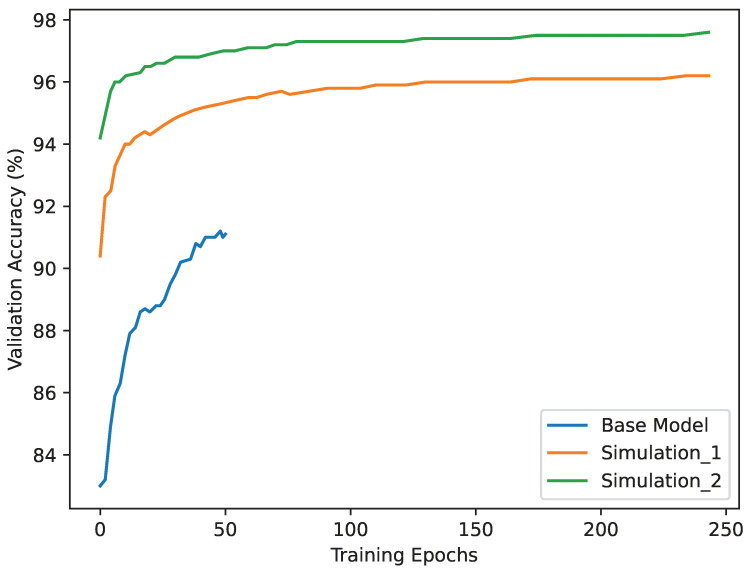
Model retraining using simulated datasets collected during the live trials.

Following each simulation exercise, the AI model was retrained using the newly collected data. The retraining process involved incorporating the original training data with the data collected from Simulation 1 and subsequently with the data from both simulation exercises. The additional data from simulations were randomly incorporated into the original dataset, ensuring a balanced representation of scenarios. To prepare for retraining and avoid overfitting, the combined final dataset was then split randomly into 75/25 proportions, with 75% used for model training and 25% held out for validation. The goal was to continuously improve the model’s accuracy and performance as more diverse and relevant data became available. The impact of the retraining process was evident in the model’s validation accuracy. Figure 8 depicted the evolution of accuracy with different training datasets. Augmenting the training data with the simulation data resulted in a noticeable improvement in the model’s validation accuracy. The use of a complete dataset that included data from both simulations resulted in higher accuracy compared to using only the dataset collected after Simulation 1 or relying solely on the original data. The iterative retraining approach was instrumental in fine-tuning the AI model’s behaviour recognition capabilities, reducing false positives (FPs), and enhancing its overall performance in detecting fight incidents in the transportation network.

### 5.4. On-Site Evaluation

The developed solution was deployed at Wollongong Train Station for its real-world functionality and performance evaluation. In evaluating the performance of our AI models, we used several key evaluation metrics to assess their effectiveness in correctly classifying positive and negative instances. These metrics include accuracy, which measures the proportion of correctly classified instances among all instances; TP rate (Recall), which indicates the model’s ability to correctly identify positive cases; TN rate, which reflects the model’s success in identifying negative cases; FP rate, which measures the likelihood of incorrectly classifying negative instances as positive; and false-negative (FN) rate, which represents the likelihood of incorrectly classifying positive instances as negative. Additionally, we calculated precision, which evaluates the proportion of true-positive predictions among all positive predictions made by the model, and F1 score, the harmonic mean of precision and recall, providing a balance between the two for cases where both FPs and FNs are critical. These metrics collectively offer a comprehensive view of the model’s performance across various aspects of classification.

During the 8-week test period at Wollongong Train Station, the AI system generated a total of 3600 alerts based on its detection of potential fight incidents. Out of these numerous alerts, only 36 were confirmed as actual fight incidents. Among the confirmed incidents, the AI correctly identified 30 as true positives, indicating instances where fights were accurately detected. However, there were six incidents that were missed by the AI, resulting in FNs, which means the AI failed to identify those fights. The initial FP rate observed during the live trial was relatively high, with the AI generating up to one FP per CCTV second. However, continuous updates and retraining of the AI model led to improvements in the actual FP rate after each iteration. Specifically, the first update of the model, incorporating the original data along with data from the first simulation exercise, demonstrated substantial enhancements in key performance indicators. For instance, accuracy, true negative rate, and FP rate were notably improved in events collected for a week after the deployment of the updated model (Table 1). The primary objective was to achieve a low FP rate, essential for ensuring the reliability and operational effectiveness of the solution.

**Figure 9:**
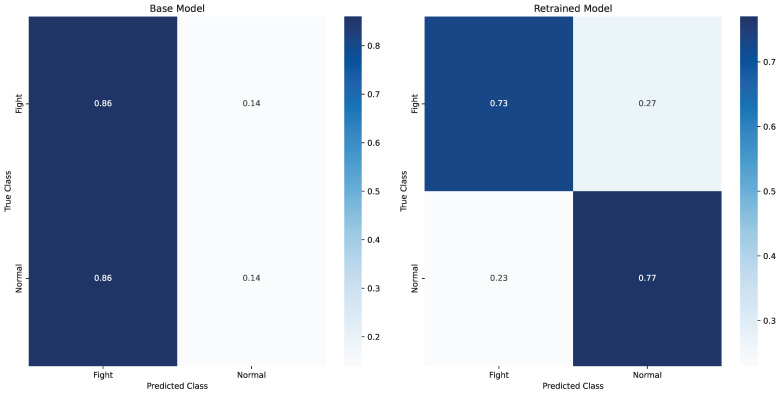
Confusion matrices for the base and retrained models.

The base model exhibited relatively poor overall performance, with an accuracy of only 0.16, which indicates that the model struggled to differentiate between the “Fight” and “Normal” classes (see Table 1). This low accuracy suggests a substantial misclassification rate, which is further reflected in the extreme imbalance between the TP and TN (see Figure 9). While the TP for the base model is 0.86, indicating a strong ability to identify the positive class (Fight), the TN is alarmingly low at 0.14, which implies that the model failed to recognize the negative class (Normal) effectively. This imbalance is compounded by a FP of 0.86, suggesting that the model frequently misclassified “Normal” instances as “Fight”. The FN, while low at 0.14, does not fully compensate for the high number of FP, which is a critical drawback for practical applications where FP can have significant consequences.

In contrast, the retrained model demonstrated substantial improvements across most metrics. The accuracy increased to 0.77, which indicates a marked enhancement in the model’s ability to correctly classify both positive (Fight) and negative (Normal) instances. The retrained model’s improved performance is particularly evident in its TN of 0.77, which indicates a much better recognition of the negative class compared to the base model. This reduction in FP to 0.23 suggests that the retrained model is more reliable and accurate when distinguishing between the two classes. Additionally, the precision for the retrained model improved to 0.76, reflecting a much stronger ability to predict the positive class correctly. This, in turn, resulted in an improved F1 score of 0.74, signalling a better balance between precision and recall. However, it is worth noting that the retrained model’s TP decreased to 0.73, slightly lower than the 0.86 observed in the base model. This decrease in recall suggests that while the retrained model is more effective at minimizing FPs, it does so at the cost of missing some true-positive instances. This trade-off highlights a common challenge in machine learning: the balancing act between precision and recall.

**Figure 10:**
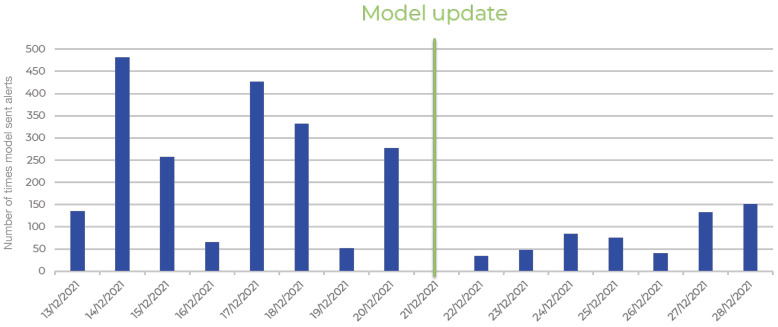
Field evaluation of the ai model in terms of false positives (FPs) (the green line represents the time when weights of the model were updated with latest training iteration).

Overall, the retrained model outperformed the base model in several key metrics, including accuracy, precision, and F1 score. While there is a slight sacrifice in recall, the retrained model’s overall performance is far more reliable and balanced (see Table 1 and Figure 9), making it better suited for deployment in practical scenarios where both positive and negative class detection are critical. The improvements in the retrained model reflect the positive impact of additional data and retraining, suggesting that further refinement with additional fine-tuning or more diverse datasets could yield even better performance. This final model, deployed on the 21st of December, successfully lowered the FP rate to between 3 and 12 per hour per CCTV feed, representing a considerable reduction. Notably, this progress was achieved despite conducting only two simulation exercises to gather additional data (see Figure 10).

**Figure 11:**
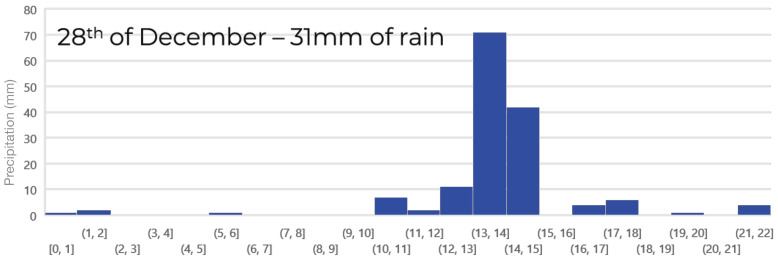
Field evaluation of the AI model in terms of false positives (FPs) for a rainy day.

An interesting finding during the live test was the impact of rainy weather on the FP rate. The day with the highest number of FPs (152) occurred on the 28 December, coinciding with heavy rainfall of 31 mm, as reported by the Bureau of Meteorology. Figure 11 illustrates the distribution of the FP rate per hour for that particular day, revealing that the majority of FPs occurred during the daytime, with a peak between 1 pm and 3 pm.

The influence of rainy weather on FPs was attributed to the scarcity of training data that depicted incidents occurring during such weather conditions. Rain introduced significant noise and dynamic elements into the captured video feed, which impacted the AI’s predictions, leading to a higher FP rate. However, the AI’s performance remained unaffected by low light conditions at night, as only a few FPs were detected during night-time. This resilience was attributed to the diverse range of luminosity present in the training data, with some video clips recorded during night time or in black and white.

The on-site evaluation demonstrated the AI approach’s potential for real-time incident detection and its ability to aid transportation operators in responding promptly to incidents. The successful application at Wollongong Train Station indicates the scalability of the solution, making it feasible for extension to other locations and adaptability to different types of behaviours, provided sufficient data are available for retraining the AI. An additional advantage of the deployed model is its ability to be continuously retrained and improved. User assessments of the AI’s predictions through the developed web application played a vital role in enhancing its accuracy and fine-tuning its behaviour recognition capabilities in real-world scenarios.

## 6. Challenges and Future Research Directions

The development a smart sensing video-based action recognition pipeline involves several challenges. One of the major hurdles was obtaining ethics clearance and legal permissions to access live video feeds from security cameras at Wollongong Train Station. Ensuring compliance with privacy regulations and obtaining the necessary approvals for data collection was a time-consuming and resource-intensive process. Moreover, the COVID-19 pandemic posed significant impacts on the project. UOW’s restrictions on student involvement in simulations and New South Wales (NSW) Health Orders preventing in-person simulations led to delays in the project timeline. Additionally, acquiring sufficient training data to teach the AI to recognize a specific behaviour proved to be a challenge, prompting the project team to organize simulation exercises in collaboration with Transport for NSW and Sydney Trains. Deploying AI also resulted in a high number of FPs, which needs to be addressed before it can be operationalized by Sydney Trains. While the AI showed consistent performance across indoor and outdoor settings and different times of the day, it struggled with rainy weather, indicating the need for more training data in such conditions.

Several future research directions have been identified to enhance the effectiveness of the developed AI-based surveillance system. First and foremost, improving the accuracy of the current model requires continuous data collection, especially in challenging weather conditions like rainy weather. In real-world scenarios with complex conditions, such as low light, rainy weather, or high crowd density, a higher rate of FPs is expected. While not ideal, this is preferable to having high FNs, as it prioritizes safety by ensuring potentially unsafe behaviours are detected. These FPs will be systematically added to the training dataset, providing valuable data to improve model performance in future deployments. By collecting this automatically annotated data, the model can be retrained periodically, with each iteration enhancing its accuracy and reliability in challenging environments. Overall, continuous training and access to more data are expected to improve the AI’s performance over time, gradually reducing FPs and achieving human-level accuracy. The primary aim of this study was to develop a lightweight, operational pipeline capable of real-time monitoring on edge computing infrastructure, rather than to achieve the absolute highest accuracy in action recognition. However, future research may explore integrating more advanced models, such as transformer-based action recognition models (e.g., action transformer (AcT) [26]) or visual language models (VLMs) (e.g., VILA [27]). Moreover, exploring a model based on pose estimation, using 3D skeletons to capture subtle behaviours, presents a promising direction. This approach would require a new annotation exercise for the collected dataset and has the potential to enhance the AI’s ability to detect more complex actions. To fully leverage GPUs for efficient decoding and AI computations, an updated version of the inference pipeline can be developed using a complete DeepStream implementation instead of the current one based on ONNXRuntime and TensorRT.

While this study successfully demonstrated the feasibility and effectiveness of a real-time AIoT solution for monitoring unsafe behaviours, it did so under the assumption of continuous video feed availability. Future research could extend the system’s resilience to handle real-world operational challenges, such as video stream interruptions or network failures. Potential enhancements include incorporating local buffering on edge devices, on-device action recognition for brief outages, and multi-path streaming to alternate servers for redundancy. Additionally, incorporating automatic reconnection protocols and cloud failover instances could further enhance the robustness and reliability of the system.

## 7. Conclusions

In this study, we developed an advanced AI solution to detect violence and unsafe behaviours in public transport systems, particularly trains. The TPN action recognition model was trained and deployed to identify fighting and normal behaviours in the CCTV streams from train station. The proposed solution was deployed over local server where AI inference was carried out using the ONNXRuntime and inference results were transmitted to a web application using MQTT. The base model was deployed at Wollongong Train Station, and simulated data were collected which helped in improving the model training performance to 97% from 94%. The final solution with the retrained model was tested for eight weeks as a field testing and was able to achieve the reliable FP rate of 23% and correctly identified 30 true-positive incidents. However, six FNs were observed, indicating the need for further improvement, especially in recognizing incidents during adverse weather conditions. Continuous retraining of the AI model played a vital role in refining its behaviour recognition capabilities and reducing FPs. The success of the deployment showcased the solution’s scalability and adaptability, making it applicable to other locations and behaviours with sufficient data for retraining.

## Figures and Tables

**Table 1:** On-site evaluation of the retrained ai model.

	Accuracy	TP Rate	TN Rate	FP Rate	FN Rate	Precision	Recall	F1 Score
Base Model	0.16	0.86	0.14	0.86	0.14	0.5	0.86	0.64
Retrained with Sim2 Data	0.77	0.73	0.77	0.23	0.27	0.76	0.73	0.74

## Data Availability

Data are contained within the article.

## Abbreviations

The following abbreviations are used in this manuscript:

AWS	Amazon Web Service
CCTV	Closed Circuit Television
TPN	Temporal Pyramid Network
AI	Artificial Intelligence
RQ	Research Question
GPU	Graphical Processing Unit
3D	Three-Dimensional
GRU	Graph Recurrent Unit
2D	Two-Dimensional
FPS	Frames per Second
TP	True Positive
TN	true negative
UOW	University of Wollongong
FP	False Positive
FN	False Negative
NSW	New South Wales
